# Short-Term Safety of Gastric Endoscopic Submucosal Dissection in Patients Aged 80 Years or Older Under a Standardized Inpatient Post-Procedural Surveillance Pathway: Data from a 13-Year Real-World Cohort Study

**DOI:** 10.3390/jcm15197373

**Published:** 2026-09-23

**Authors:** Youn I Choi, Sun Young Won, Yong Suk Cho, Byung Kyu Park, Chan Su Park, Jong Won Choi, Chun Kyon Lee

**Affiliations:** Division of Gastroenterology and Hepatology, Department of Internal Medicine, National Health Insurance Service Ilsan Hospital, 100 Ilsan-ro, Ilsandong-gu, Goyang 10444, Republic of Korea; sywon@nhimc.or.kr (S.Y.W.); cho6706@nhimc.or.kr (Y.S.C.); bkpark@nhimc.or.kr (B.K.P.); wpakcs@nhimc.or.kr (C.S.P.); nicewon@nhimc.or.kr (J.W.C.); cklee33@nhimc.or.kr (C.K.L.)

**Keywords:** gastric neoplasia, endoscopic submucosal dissection, aged 80 and over, safety, peri-procedural surveillance, gastric cancer

## Abstract

**Background/Objectives:** Gastric endoscopic submucosal dissection (ESD) is an established organ-preserving treatment for gastric neoplasia, but the short-term safety of ESD in patients aged 80 years or older remains clinically important. We evaluated the efficacy and short-term safety of gastric ESD in patients aged ≥80 years treated under a standardized inpatient post-procedural surveillance pathway. **Methods:** This retrospective cohort study included 2021 consecutive unique patients who underwent gastric ESD for gastric adenoma or gastric cancer at a single institution between April 2013 and March 2026. Patients were classified as <80 years or ≥80 years. Standard hospitalization was 3 nights/4 days for patients not receiving antithrombotic agents and 5 nights/6 days for those receiving antithrombotic agents. The primary safety outcomes were post-ESD bleeding, perforation, pneumonia, and delirium. Absolute risk differences with 95% confidence intervals (CIs) were calculated, and multivariable logistic regression was performed to evaluate the association between age ≥ 80 years and selected efficacy and safety outcomes. **Results:** Among the 2021 patients, 285 were aged ≥80 years and 1736 were aged <80 years. Non-curative resection was more frequent in patients aged ≥80 years (7.7% vs. 4.6%; *p* = 0.026), but this difference was not significant after lesion-adjusted analysis. Post-ESD bleeding (2.5% vs. 2.4%), perforation (0.4% vs. 0.7%), and pneumonia (0% vs. 0.1%) were comparable between groups, while delirium occurred in one patient aged ≥80 years. No 30-day mortality occurred in either group. In multivariable analysis, age ≥ 80 years was not independently associated with post-ESD bleeding or perforation, and the primary safety findings were similar after propensity-score matching. **Conclusions:** In this single-center cohort, gastric ESD in selected patients aged ≥80 years was not independently associated with non-curative resection or increased short-term adverse outcomes. Chronological age alone should not preclude gastric ESD when standardized monitoring and individualized risk assessment are applied.

## 1. Introduction

Gastric cancer remains a major cause of cancer-related mortality worldwide [1,2]. As life expectancy increases, gastroenterologists are increasingly faced with treatment decisions for elderly patients with gastric neoplasia [3,4]. In this population, surgical resection may be limited by reduced physiologic reserve, multiple comorbidities, and concerns regarding postoperative recovery [5,6,7,8,9,10].

Gastric endoscopic submucosal dissection (ESD) is an established treatment for superficial gastric neoplasia, allowing en bloc resection and accurate histopathologic assessment while preserving the stomach [11,12,13,14,15,16,17,18,19]. These advantages are particularly relevant in elderly patients, in whom treatment decisions must also consider comorbidity burden, cardiopulmonary reserve, antithrombotic therapy, and competing risks of non-cancer mortality [20,21,22,23,24,25,26]. Although previous studies have reported acceptable technical outcomes in older patients, concerns remain regarding post-ESD adverse events. In addition to bleeding and perforation, respiratory complications, delayed oral intake, prolonged hospitalization, delirium, unplanned hospital visits, readmission, and short-term mortality are clinically relevant in this population [21,27,28,29,30,31,32,33,34].

Previous studies have investigated the safety of gastric ESD in elderly patients, but the available evidence remains heterogeneous with respect to age thresholds, sample size, and definitions of safety outcomes. Studies in patients aged ≥75 years have generally reported preserved en bloc and complete resection rates and complication rates comparable to those in younger patients [17]. However, respiratory complications remain an important concern. Akasaka et al. reported a higher rate of pneumonia in patients aged ≥75 years than in younger patients (3.3% vs. 0.5%, *p* < 0.01), while Toyokawa et al. observed numerically higher aspiration pneumonia and significantly higher delayed bleeding rates in elderly patients [27,35]. In studies focusing on patients aged ≥80 or ≥85 years, technical outcomes were generally maintained, although non-bleeding adverse events, including perforation and pneumonia, remained clinically relevant [25,33].

Despite these findings, uncertainty remains regarding short-term morbidity, delirium, hospitalization, and healthcare utilization in very elderly patients undergoing gastric ESD. Previous studies have often compared age groups without fully accounting for differences in post-procedural surveillance, hospitalization practices, and antithrombotic management. Evaluating younger and older patients within the same institutional setting and under a standardized inpatient post-ESD surveillance pathway may therefore provide a more consistent assessment of the clinical relevance of advanced age. Accordingly, we evaluated the efficacy and short-term safety of gastric ESD in patients aged ≥80 years compared with those aged <80 years in a large single-center cohort managed under a standardized inpatient post-ESD surveillance pathway.

## 2. Materials and Methods

### 2.1. Study Design and Population

This retrospective cohort study included consecutive patients who underwent gastric ESD for gastric adenoma or gastric cancer at our institution between April 2013 and March 2026. Each patient contributed one index gastric lesion and one ESD procedure. For patients who underwent more than one gastric ESD during the study period, only the first eligible ESD was included, and subsequent ESD procedures were excluded. A total of 2021 patients were included in the final analysis. Eligible patients were classified into two groups according to age at the time of ESD: patients aged ≥80 years and those aged <80 years. Clinical, endoscopic, and pathologic data were retrospectively collected from the electronic medical records and endoscopy database. Gastric epithelial neoplasia was classified into low-grade adenoma, high-grade adenoma, and early gastric cancer according to the final pathologic diagnosis after ESD.

The study protocol was approved by the Institutional Review Board of the National Health Insurance Service Ilsan Hospital (IRB No. NHIMC 2026-03-010; approval date, 26 March 2026). Written informed consent for the ESD procedure was obtained as part of routine clinical care. The requirement for written informed consent for participation in this retrospective study was waived by the Institutional Review Board.

### 2.2. ESD Procedure and Peri-Procedural Care

All ESD procedures were performed by five staff endoscopists who routinely performed gastric ESD at our institution. The indication for ESD was determined from the pre-procedural biopsy, endoscopic appearance, lesion size and location, and expected depth of invasion. Chronological age alone was not considered a contraindication to ESD. In patients aged ≥80 years, the decision to perform ESD was individualized according to lesion characteristics, comorbidity burden, overall clinical condition, anticipated procedural tolerance, and patient preference.

Sedation was individualized by endoscopists, and was not governed by a single fixed drug-and-dose protocol throughout the 13-year study period. Among the five endoscopists, four used midazolam alone for both the induction and maintenance of sedation, whereas one used midazolam-based sedation with adjunctive propofol as needed. Propofol monotherapy was not used. During ESD, continuous electrocardiographic and pulse oximetry monitoring was performed, and blood pressure was measured at 5-min intervals.

Patients were managed according to a standardized inpatient post-ESD surveillance pathway. Continuous intravenous proton pump inhibitor (PPI) therapy was initiated on the day of ESD, followed by oral PPI therapy for 2 months after discharge. Water intake and oral diet were resumed on the morning after ESD. In general, patients who were not receiving antithrombotic agents were admitted for 3 nights and 4 days, whereas patients receiving antithrombotic agents were generally observed for 5 nights and 6 days.

Periprocedural management of antithrombotic agents was individualized according to contemporaneous guideline recommendations and the patient’s bleeding and thromboembolic risks. Decisions regarding temporary interruption, heparin bridging, and post-ESD resumption were made in consultation with the relevant specialists, including cardiologists, neurologists, or other treating physicians, as appropriate. For antithrombotic agents that were temporarily interrupted, therapy was resumed as early as clinically feasible after adequate hemostasis had been confirmed, generally within 48 h after ESD. The exact timing of resumption was individualized according to the type of antithrombotic agent and the patient’s thromboembolic and bleeding risks.

After ESD, patients were monitored for abdominal symptoms, fever, bleeding, respiratory symptoms, and changes in mental status. Chest radiography and laboratory tests were routinely performed daily during the standard 3-night, 4-day hospitalization period according to the institutional post-ESD care protocol. Routine post-ESD endoscopy was not performed. Hospitalization was prolonged beyond the standard hospitalization period when post-ESD complications, including bleeding, perforation, delirium, or pneumonia, were suspected or identified.

### 2.3. Data Collection

Clinical, endoscopic, and pathologic data were retrospectively collected and reviewed for all included patients. Clinical variables included age, sex, Charlson Comorbidity Index (CCI), antithrombotic-agent use, and duration of hospitalization. Endoscopic and pathologic variables included lesion location, lesion size, gross morphology, histologic type, depth of invasion, resection-margin status, lymphovascular and venous invasion, perineural invasion, and final pathologic diagnosis. Final pathology was classified as low-grade adenoma, high-grade adenoma, or EGC.

Lesion location was categorized as the antrum/lower third, body/middle third, fundus/cardia/upper third, or angle/incisura. Depth of invasion was determined from the final histopathologic specimen and classified as low-grade dysplasia, high-grade dysplasia, mucosal invasion (pT1a), submucosal invasion <500 μm, or submucosal invasion ≥500 μm. Resection margins were classified as negative or involved according to the final histopathologic assessment.

### 2.4. Outcome Definitions

The primary efficacy outcome was non-curative resection. Non-curative resection was determined from the final histopathologic findings according to the curability criteria applicable to gastric ESD. Additional surgery performed after ESD was evaluated descriptively among patients with non-curative resection.

The primary safety outcomes were post-ESD bleeding, perforation, pneumonia, and delirium. Post-ESD bleeding was defined as clinically significant bleeding requiring endoscopic hemostasis, transfusion, or another therapeutic intervention. Perforation was defined as an endoscopically identified mural defect or the presence of free air attributable to the ESD procedure. Post-ESD pneumonia was defined as new respiratory symptoms and/or clinical signs developing after ESD, together with a new pulmonary infiltrate on chest radiography requiring antibiotic treatment and continued hospitalization beyond routine post-ESD observation. Post-ESD delirium was defined as an acute documented change in mental status during the post-procedural period.

Secondary safety and healthcare-utilization outcomes included an emergency department visit within 7 days, ESD-related readmission within 30 days, any hospitalization within 30 days, complication-related repeat endoscopy within 15 days, complication-related emergency surgery within 15 days, a 30-day composite safety and healthcare-utilization outcome, and 30-day mortality. ESD-related readmission was defined as hospitalization for an ESD-related complication. Any hospitalization within 30 days included both ESD-related readmission and hospitalization for reasons unrelated to ESD and was therefore considered a descriptive healthcare-utilization outcome.

Primary and secondary outcomes were assessed independently, and individual patients could experience more than one outcome. For the composite outcome, however, each patient was counted only once regardless of the number of component events. The composite outcome was defined as the occurrence of at least one of the following: post-ESD bleeding, perforation, pneumonia, or delirium; an emergency department visit within 7 days; any hospitalization within 30 days; complication-related repeat endoscopy or emergency surgery within 15 days; or death within 30 days. Planned oncologic surgery after non-curative ESD was not considered a safety event.

### 2.5. Statistical Analysis

Continuous variables are expressed as mean ± standard deviation or median with interquartile range. Categorical variables are expressed as number and percentage. The <80-year and ≥80-year groups were compared using the Student’s t test or Mann–Whitney U test for continuous variables and the chi-square test or Fisher’s exact test for categorical variables, as appropriate.

For binary efficacy and safety outcomes, absolute risk differences were calculated as the event rate in patients aged ≥80 years minus that in patients aged <80 years, with corresponding 95% confidence intervals (CIs).

Multivariable logistic regression analyses were used to evaluate the association between age ≥ 80 years and selected efficacy and safety outcomes, with age < 80 years as the reference category. Base models were adjusted for sex, CCI, and antithrombotic-agent use. Multivariable analysis of non-curative resection was restricted to patients with EGC. Additional surgery performed after ESD was analyzed descriptively among patients with non-curative resection because of the small number of events. Lesion-adjusted sensitivity analyses were performed to account for differences in lesion characteristics. For the efficacy outcome, the EGC-restricted model was additionally adjusted for lesion size, lesion location, depth of invasion, and calendar period. For safety outcomes, lesion-adjusted models were performed in the overall cohort and were additionally adjusted for pathology category, lesion size, lesion location, and calendar period. Calendar period was categorized as April 2013–December 2019 and January 2020–March 2026 to account for potential temporal changes in ESD practice.

As a sensitivity analysis, 1:1 propensity-score matching was performed between patients aged ≥80 years and those aged <80 years using sex, CCI, antithrombotic-agent use, final pathology category, and calendar period. Covariate balance after matching was assessed using absolute standardized mean differences, with values <0.10 considered indicative of acceptable balance.

Multivariable analyses were not performed for post-ESD pneumonia or delirium because only two and one events, respectively, occurred in the entire cohort, resulting in insufficient events for reliable adjusted effect estimation.

Results are presented as adjusted odds ratios (ORs) with 95% confidence intervals (CIs). A two-sided *p* value < 0.05 was considered statistically significant. Statistical analyses were performed using IBM SPSS Statistics version 31.0 (IBM Corp., Armonk, NY, USA).

### 2.6. Use of Artificial Intelligence

Generative artificial intelligence developed by OpenAI (ChatGPT, GPT-4; OpenAI, San Francisco, CA, USA) was used solely to assist with English-language expression and wording during manuscript preparation and revision. All scientific content, analyses, interpretations, and conclusions were reviewed and verified by the authors.

## 3. Results

### 3.1. Study Population

The study flow is shown in Figure 1. Between April 2013 and March 2026, a total of 2021 patients who underwent one index gastric ESD procedure for gastric adenoma or gastric cancer were included in the analysis. Among them, 285 patients were aged ≥80 years and 1736 patients were aged <80 years.

### 3.2. Baseline Characteristics

Patients aged ≥80 years had a greater comorbidity burden than younger patients (Table 1). The mean CCI was higher (2.6 ± 1.8 vs. 1.8 ± 1.6, *p* < 0.001), and the proportion of patients with CCI ≥3 was also higher (49.8% vs. 41.2%, *p* = 0.007) in the ≥80-year group (Table 1). Antithrombotic-agent use was numerically more frequent in the ≥80-year group than in the <80-year group (4.2% vs. 2.5%, *p* = 0.110) (Table 1). EGC accounted for a larger proportion of lesions in the older group than in the younger group (51.9% vs. 40.7%; overall *p* = 0.001) (Table 1).

### 3.3. Lesion and Pathologic Characteristics

In the overall cohort, 923 lesions (45.7%) were classified as low-grade adenoma, 243 (12.0%) as high-grade adenoma, and 855 (42.3%) as EGC (Table 2). The antrum or lower third was the most common lesion location (66.3%), followed by the body or middle third (21.9%), angle/incisura (8.9%), and fundus/cardia/upper third (3.0%) (Table 2).

A negative histologic resection margin was achieved in 98.2% of all cases (Table 2). Among the 855 patients with EGC, 25 (2.9%) had intraepithelial/CIS/HGD-only disease, 737 (86.2%) had mucosal invasion, 44 (5.1%) had submucosal invasion <500 μm, and 49 (5.7%) had submucosal invasion ≥500 μm (Table 2).

### 3.4. Efficacy Outcomes According to Age Group

Non-curative resection was more frequent in patients aged ≥80 years than in those aged <80 years (7.7% vs. 4.6%; risk difference, 3.11 percentage points; 95% CI, 0.32–6.91; *p* = 0.026). Among the patients with non-curative resection, additional surgery was performed in 2 out of 22 patients aged ≥80 years (9.1%) and 13 out of 80 patients aged <80 years (16.3%) (risk difference, −7.16 percentage points; 95% CI, −18.78 to 12.66; *p* = 0.514) (Table 3).

### 3.5. Short-Term Safety Outcomes

Post-ESD bleeding occurred in 2.5% of patients aged ≥80 years, and 2.4% of those aged <80 years (risk difference, 0.09 percentage points; 95% CI, −1.41 to 2.69; *p* = 0.923). Perforation occurred in 0.4% and 0.7%, respectively (risk difference, −0.40 percentage points; 95% CI, −1.00 to 1.24; *p* = 0.707). Two cases of post-ESD pneumonia occurred in the younger group, whereas one case of delirium occurred in the older group. No 30-day deaths were observed.

ESD-related readmission within 30 days occurred in 0.7% of older patients and 2.1% of younger patients (*p* = 0.114). The corresponding rates for any hospitalization within 30 days were 2.5% and 4.7% (*p* = 0.090). The 30-day composite safety and healthcare-utilization outcome occurred in 4.6% and 7.0%, respectively (risk difference, −2.41 percentage points; 95% CI, −4.69 to 0.87; *p* = 0.130) (Table 4).

### 3.6. Multivariable Analyses

Age ≥ 80 years was not associated with non-curative resection in the EGC-restricted base model (adjusted OR, 1.11; 95% CI, 0.48–2.61; *p* = 0.802). Age ≥ 80 years was also not associated with post-ESD bleeding (adjusted OR, 0.86; 95% CI, 0.38–1.97; *p* = 0.727) or perforation (adjusted OR, 0.38; 95% CI, 0.05–2.94; *p* = 0.352). In the base model, age ≥ 80 years was associated with lower odds of ESD-related readmission within 30 days (adjusted OR, 0.43; 95% CI, 0.19–0.95; *p* = 0.037). Multivariable estimates for pneumonia and delirium were not calculated because of the small number of events.

The lesion-adjusted analysis yielded a similar finding for non-curative resection (adjusted OR for age ≥ 80 years, 1.01; 95% CI, 0.30–3.40; *p* = 0.993). The corresponding estimates were 0.82 (95% CI, 0.33–2.05; *p* = 0.668) for post-ESD bleeding and 0.75 (95% CI, 0.17–3.31; *p* = 0.707) for perforation. The association with ESD-related readmission was no longer statistically significant after lesion adjustment (adjusted OR, 0.43; 95% CI, 0.12–1.51; *p* = 0.186) (Table 5).

After 1:1 propensity-score matching, covariate balance was acceptable, with all absolute standardized mean differences <0.10. Post-ESD bleeding, perforation, and ESD-related readmission did not differ significantly between the matched age groups. In the matched cohort, any hospitalization within 30 days occurred in 2.5% of patients aged ≥80 years and 7.0% of those aged <80 years (*p* = 0.015) (Tables S2 and S3).

In the calendar-period sensitivity analysis, no significant age-by-period interaction was observed for post-ESD bleeding or perforation. An age-by-period interaction was observed for ESD-related readmission (*p* for interaction = 0.042). However, this finding should be interpreted cautiously because of the small number of events (Table S4).

## 4. Discussion

In this 13-year real-world cohort study of 2021 patients who underwent gastric ESD for gastric adenoma or gastric cancer under a standardized inpatient post-ESD surveillance pathway, crude non-curative resection was more frequent in patients aged ≥80 years than in those aged <80 years. However, age ≥ 80 years was not independently associated with non-curative resection in the EGC-restricted lesion-adjusted analysis. The incidence of major short-term post-ESD complications was low in both groups and did not differ significantly. Delirium was rare, with only one event observed in the ≥80-year group. These findings suggest that under standardized peri-procedural surveillance, gastric ESD can be performed safely in selected patients aged ≥80 years and that chronological age alone should not be regarded as a contraindication to endoscopic treatment.

A major strength of the present study is that both age groups were treated at the same institution within a standardized inpatient post-ESD surveillance pathway. Patients not receiving antithrombotic agents were generally hospitalized for 3 nights and 4 days, whereas those receiving antithrombotic agents were generally observed for 5 nights and 6 days. Antithrombotic management itself was individualized according to contemporaneous guideline recommendations and each patient’s bleeding and thromboembolic risks, with temporarily interrupted therapy generally resumed within 48 h after ESD once adequate hemostasis had been confirmed. This common surveillance framework reduced variation in post-procedural observation while allowing clinically appropriate individualized management. In this setting, patients aged ≥80 years had short-term safety outcomes broadly comparable to those of younger patients, while delirium remained rare.

Our findings are consistent with previous studies reporting acceptable efficacy of gastric ESD in older patients. Kakushima et al. reported an en bloc R0 resection rate of 96% in patients aged ≥75 years, without a significant increase in procedure-related complications compared with younger patients [17]. Similar outcomes have been reported in patients aged ≥80 years [25,36]. In our cohort, crude non-curative resection was more frequent in patients aged ≥80 years; however, age ≥ 80 years was not associated with non-curative resection after adjustment for lesion-related factors in the EGC subgroup. Among patients with non-curative resection, the proportion undergoing additional surgery also did not differ significantly between age groups. These findings suggest that advanced age alone may not be a major determinant of short-term efficacy after gastric ESD.

The safety profile observed in our study differed in several respects from previous reports. Respiratory complications have been an important concern in elderly patients undergoing gastric ESD. Akasaka et al. reported a significantly higher pneumonia rate in patients aged ≥75 years than in younger patients (3.3% vs. 0.5%; *p* < 0.01) [35]. Toyokawa et al. reported delayed bleeding, perforation, and pneumonia rates of 9.6%, 1.7%, and 0.87%, respectively, among elderly patients undergoing gastric ESD [27]. Similarly, studies focusing on patients aged ≥80 or ≥85 years have shown preserved resection outcomes but continued concern regarding perforation and pneumonia [25,33]. These findings indicate that although gastric ESD is technically feasible in very elderly patients, non-bleeding adverse events remain clinically relevant. Our study extends these observations by evaluating delirium, hospitalization, and short-term healthcare utilization within a standardized inpatient post-ESD surveillance pathway.

Comorbidity is an important consideration when interpreting outcomes in very elderly patients. In the present cohort, patients aged ≥80 years had a higher mean CCI and were more likely to have a CCI of ≥3 than younger patients. Thus, the comparable short-term outcomes in the older group were observed despite a greater measured comorbidity burden. This does not, however, exclude the possibility of clinical selection. Information on performance status, activities of daily living, frailty, and cognitive function was not consistently available, and older patients with relatively preserved functional reserve may have been more likely to undergo ESD. These findings support an individualized approach to ESD in very elderly patients, taking overall clinical condition and expected benefit into account rather than relying on chronological age alone.

The structured inpatient surveillance pathway may also have contributed to the low incidence of delirium and other post-ESD complications observed in our cohort. Patients were routinely observed during the early post-procedural period, when bleeding, perforation-related symptoms, fever, respiratory symptoms, poor oral intake, and acute cognitive changes were most likely to become clinically apparent. This structured observation may have facilitated the early recognition and management of post-ESD adverse events. Therefore, the favorable short-term outcomes observed in this study likely reflect both careful patient selection and the institutional post-ESD surveillance pathway.

Antithrombotic therapy was an important consideration in peri-procedural management. Because ESD is a high-bleeding-risk endoscopic procedure, management of antiplatelet and anticoagulant agents was individualized according to each patient’s bleeding and thromboembolic risks. When temporary interruption was considered necessary, the timing of interruption and resumption was determined in accordance with contemporaneous guidelines, and when appropriate, after consultation with the relevant specialists. Antithrombotic therapy was generally resumed within 48 h after ESD once adequate hemostasis had been confirmed and no contraindication was present [37].

The present findings have practical implications for decision-making in elderly patients with gastric adenoma or cancer. In many clinical settings, advanced age may lead to hesitation in offering ESD, even when the lesion is technically resectable and the patient’s overall condition is acceptable. However, our data suggest that age ≥ 80 years itself was not independently associated with non-curative resection or with higher short-term post-ESD complication rates. Treatment decisions should therefore be individualized and based on functional status, comorbidity burden, cardiopulmonary reserve, antithrombotic medication use, cognitive status, lesion characteristics, and patient preference rather than chronological age alone.

This study has several limitations. First, it was a retrospective single-center study, and selection bias cannot be excluded. Patients aged ≥80 years who underwent ESD were selected after the clinical assessment of procedural feasibility and overall condition. Second, standardized measures of performance status, activities of daily living, cognitive function, and frailty were not consistently available in this retrospective cohort, which limited our ability to account for functional vulnerability beyond comorbidity burden. Although the older group had a higher measured comorbidity burden based on the CCI, patients with relatively preserved functional or physiologic reserve may have been more likely to undergo ESD. CCI was used to quantify comorbidity burden but does not capture functional reserve or frailty. Third, sedation depth may also not have been uniformly captured across the entire study period. Fourth, because the study period extended from 2013 to 2026, changes in devices, endoscopic technique, sedation practice, and post-ESD care may have influenced the outcomes. Finally, although short-term outcomes were evaluated, longer-term outcomes, including recurrence, metachronous lesions, non-cancer mortality, and quality of life, require further study.

Despite these limitations, this study included a large consecutive cohort with a substantial number of patients aged ≥80 years and evaluated outcomes relevant to this population, including delirium and short-term healthcare utilization. In addition, both age groups were managed within the same institutional inpatient post-ESD surveillance pathway, allowing short-term outcomes to be compared under similar post-procedural observation.

## 5. Conclusions

In this single-center cohort, gastric ESD in carefully selected patients aged ≥80 years treated at an experienced center under a structured inpatient post-ESD surveillance pathway was not independently associated with non-curative resection or increased short-term complications. These findings support the consideration of ESD in appropriately selected elderly patients within similar clinical settings, rather than on the basis of chronological age alone.

## Figures and Tables

**Figure 1:**
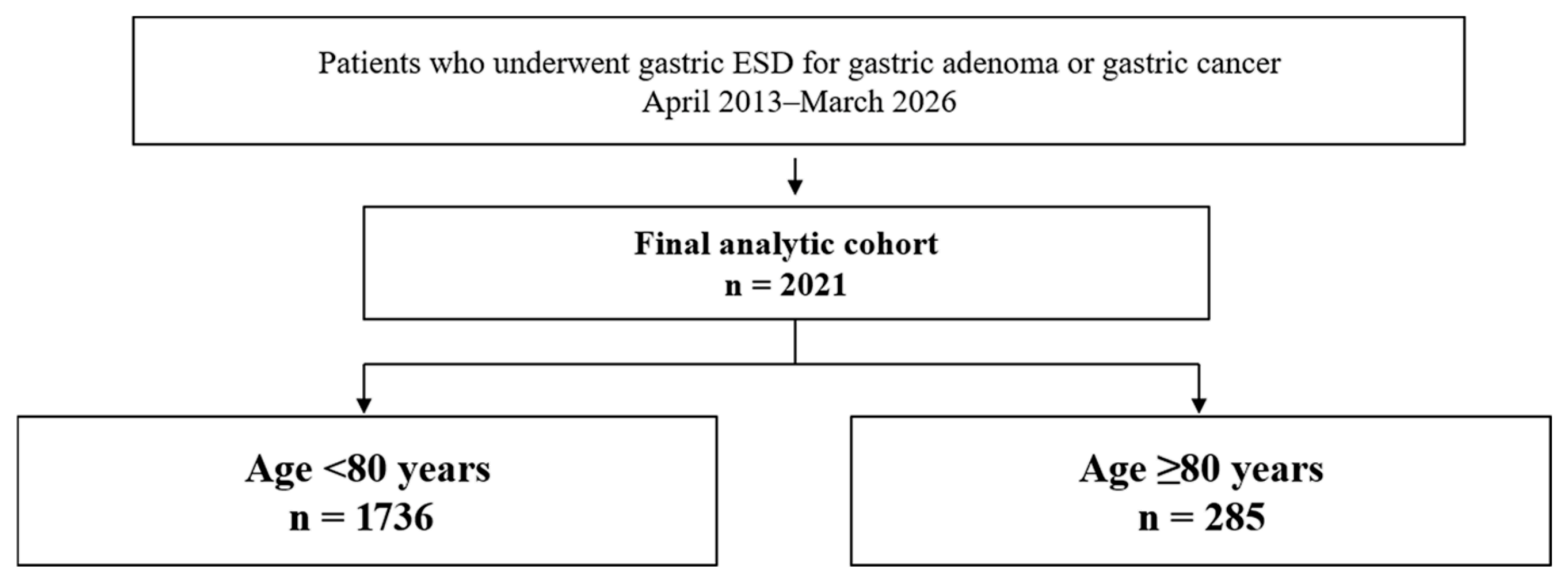
Study flow of the gastric ESD cohort.

**Table 1:** Baseline characteristics according to age group.

Characteristic	Total (*n* = 2021)	<80 Years (*n* = 1736)	≥80 Years (*n* = 285)	*p* Value
No. of patients (one lesion and one ESD per patient)	2021	1736	285	—
Age, years, mean ± SD	68.5 ± 10.1	66.1 ± 8.8	82.8 ± 2.6	<0.001
Age range, years	33–95	33–79	80–95	
Male sex, *n* (%)	1347 (66.7)	1170 (67.4)	177 (62.1)	0.079
CCI, mean ± SD	2.0 ± 1.7	1.8 ± 1.6	2.6 ± 1.8	<0.001
CCI ≥ 3, *n/N* (%)	858/2021 (42.5)	716/1736 (41.2)	142/285 (49.8)	0.007
Antithrombotic-agent use, *n* (%)	56 (2.8)	44 (2.5)	12 (4.2)	0.110
Hospital stay, days, median (IQR)	4.0 (4.0–5.0)	4.0 (4.0–4.0)	4.0 (4.0–5.0)	<0.001
Final pathology category				0.001
Low-grade adenoma	923 (45.7)	820 (47.2)	103 (36.1)	
High-grade adenoma	243 (12.0)	209 (12.0)	34 (11.9)	
Early gastric cancer	855 (42.3)	707 (40.7)	148 (51.9)	

CCI, Charlson Comorbidity Index; ESD, endoscopic submucosal dissection; IQR, interquartile range; SD, standard deviation.

**Table 2:** Lesion and pathologic characteristics according to final pathology category.

Characteristic	Total (*n* = 2021)	Low-Grade Adenoma (*n* = 923)	High-Grade Adenoma (*n* = 243)	EGC (*n* = 855)	*p* Value
**Location**					
Antrum/lower third	1340 (66.3)	585 (63.4)	160 (65.8)	595 (69.6)	0.001
Body/middle third	442 (21.9)	232 (25.1)	52 (21.4)	158 (18.5)	
Fundus/cardia/upper third	60 (3.0)	17 (1.8)	7 (2.9)	36 (4.2)	
Angle/incisura	179 (8.9)	89 (9.6)	24 (9.9)	66 (7.7)	
**Tumor size, cm, median (IQR)**	1.2 (0.8–1.8)	1.0 (0.7–1.5)	1.1 (0.7–1.5)	1.3 (0.9–2.0)	<0.001
**Gross type**					
Elevated	127 (6.3)	107 (11.6)	19 (7.8)	1 (0.1)	
Flat	49 (2.4)	39 (4.2)	10 (4.1)	0 (0.0)	
Depressed	38 (1.9)	25 (2.7)	12 (4.9)	1 (0.1)	
Elevated/IIa	1615 (79.9)	752 (81.5)	202 (83.1)	661 (77.3)	
Flat/IIb	46 (2.3)	0 (0.0)	0 (0.0)	46 (5.4)	
Depressed/IIc	146 (7.2)	0 (0.0)	0 (0.0)	146 (17.1)	
**Histologic type**					
Adenoma, low grade	923 (45.7)	923 (100.0)	0 (0.0)	0 (0.0)	
Adenoma, high grade	243 (12.0)	0 (0.0)	243 (100.0)	0 (0.0)	
Tubular adenocarcinoma, well differentiated	650 (32.2)	0 (0.0)	0 (0.0)	650 (76.0)	<0.001
Tubular adenocarcinoma, moderately differentiated	166 (8.2)	0 (0.0)	0 (0.0)	166 (19.4)	
Poorly differentiated adenocarcinoma	19 (0.9)	0 (0.0)	0 (0.0)	19 (2.2)	
Signet ring cell carcinoma	20 (1.0)	0 (0.0)	0 (0.0)	20 (2.3)	
**Lauren type**					
Intestinal				837 (97.9)	
Diffuse				12 (1.4)	
Mixed				6 (0.7)	
**Depth of invasion**					
Intraepithelial/CIS/HGD only	1191 (58.9)	923 (100.0)	243 (100.0)	25 (2.9)	<0.001
Mucosal invasion (pT1a)	737 (36.5)	0 (0.0)	0 (0.0)	737 (86.2)	
Submucosal invasion <500 μm	44 (2.2)	0 (0.0)	0 (0.0)	44 (5.1)	
Submucosal invasion ≥500 μm	49 (2.4)	0 (0.0)	0 (0.0)	49 (5.7)	
**Resection margin**					
free margin	1984 (98.2)	915 (99.1)	236 (97.1)	833 (97.4)	0.012
involved or dysplasia at margin	37 (1.8)	8 (0.9)	7 (2.9)	22 (2.6)	
Lymphovascular invasion present, *n* (%)	23 (1.1)	0 (0.0)	0 (0.0)	23 (2.7)	—
Venous invasion present, *n* (%)	1 (0.0)	0 (0.0)	0 (0.0)	1 (0.1)	—
Perineural invasion present, *n* (%)	0 (0.0)	0 (0.0)	0 (0.0)	0 (0.0)	—

Lauren classification was evaluated only in patients with EGC. EGC, early gastric cancer; IQR, interquartile range.

**Table 3:** Efficacy outcomes after gastric ESD according to age group.

Outcome	All (*n* = 2021)	<80 Years (*n* = 1736)	≥80 Years (*n* = 285)	Risk Difference, % (95% CI)	*p* Value
Non-curative resection	102/2021 (5.0)	80/1736 (4.6)	22/285 (7.7)	3.11 (0.32 to 6.91)	0.026
Additional surgery performed after ESD among patients with non-curative resection	15/102 (14.7)	13/80 (16.3)	2/22 (9.1)	−7.16 (−18.78 to 12.66)	0.514

ESD, endoscopic submucosal dissection.

**Table 4:** Short-term safety outcomes after gastric ESD according to age group.

Outcome	All (*n* = 2021)	<80 Years (*n* = 1736)	≥80 Years (*n* = 285)	Risk Difference, % (95% CI)	*p* Value
**Primary safety outcomes**					
Post-ESD bleeding	48/2021 (2.4)	41/1736 (2.4)	7/285 (2.5)	0.09 (−1.41 to 2.69)	0.923
Post-ESD perforation	14/2021 (0.7)	13/1736 (0.7)	1/285 (0.4)	−0.40 (−1.00 to 1.24)	0.707
Post-ESD pneumonia	2/2021 (0.1)	2/1736 (0.1)	0/285 (0.0)	−0.12 (−0.42 to 1.22)	1.000
Post-ESD delirium	1/2021 (0.0)	0/1736 (0.0)	1/285 (0.4)	0.35 (−0.01 to 1.96)	0.141
**Secondary safety and healthcare-utilization outcomes**					
Emergency department visit within 7 days	8/2021 (0.4)	8/1736 (0.5)	0/285 (0.0)	−0.46 (−0.91 to 0.89)	0.610
ESD-related readmission within 30 days	38/2021 (1.9)	36/1736 (2.1)	2/285 (0.7)	−1.37 (−2.31 to 0.54)	0.114
Any hospitalization within 30 days *	88/2021 (4.4)	81/1736 (4.7)	7/285 (2.5)	−2.21 (−3.88 to 0.47)	0.090
Complication-related repeat endoscopy within 15 days	50/2021 (2.5)	42/1736 (2.4)	8/285 (2.8)	0.39 (−1.22 to 3.09)	0.696
Complication-related emergency surgery within 15 days	3/2021 (0.1)	3/1736 (0.2)	0/285 (0.0)	−0.17 (−0.51 to 1.16)	1.000
30-day composite safety and healthcare-utilization outcome †	134/2021 (6.6)	121/1736 (7.0)	13/285 (4.6)	−2.41 (−4.69 to 0.87)	0.130
30-day mortality	0/2021 (0.0)	0/1736 (0.0)	0/285 (0.0)	0.00 (−0.22 to 1.33)	—

Data are presented as *n*/*N* (%). Risk differences were calculated as the percentage in patients aged ≥80 years minus that in patients aged <80 years, with 95% CIs. Primary and secondary outcomes were assessed independently, and patients could experience more than one outcome. * Any hospitalization within 30 days includes both ESD-related readmission and hospitalization unrelated to ESD and is presented descriptively. † The composite outcome was defined as the occurrence of at least one of the following: post-ESD bleeding, perforation, pneumonia, or delirium; emergency department visit within 7 days; any hospitalization within 30 days; complication-related repeat endoscopy or emergency surgery within 15 days; or death within 30 days. Each patient was counted once in the composite. Planned oncologic surgery after non-curative ESD was not considered an event. CI, confidence interval; ESD, endoscopic submucosal dissection.

**Table 5:** Multivariable analyses of the association between age ≥ 80 years and efficacy and safety outcomes after gastric ESD.

**A. Base Multivariable Models**		
**Outcome**	**Adjusted OR (95% CI) for Age ≥ 80 Years**	***p* Value**
***Efficacy outcome***		
Non-curative resection: EGC-restricted	1.11 (0.48–2.61)	0.802
***Safety outcomes***		
Post-ESD bleeding	0.86 (0.38–1.97)	0.727
Post-ESD perforation	0.38 (0.05–2.94)	0.352
Post-ESD pneumonia	Not estimable ‡	
Post-ESD delirium	Not estimable ‡	
ESD-related readmission within 30 days	0.43 (0.19–0.95)	0.037
**B. Lesion-Adjusted Sensitivity Analyses**		
**Outcome**	**Adjusted OR (95% CI) for Age ≥ 80 Years**	***p* Value**
***Efficacy outcome***		
Non-curative resection: EGC-restricted	1.01 (0.30–3.40)	0.993
***Safety outcomes***		
Post-ESD bleeding	0.82 (0.33–2.05)	0.668
Post-ESD perforation	0.75 (0.17–3.31)	0.707
Post-ESD pneumonia	Not estimable ‡	
Post-ESD delirium	Not estimable ‡	
ESD-related readmission within 30 days	0.43 (0.12–1.51)	0.186

Values are adjusted ORs (95% CIs) for age ≥ 80 years versus <80 years. Base models were adjusted for sex, CCI, and antithrombotic-agent use. For the EGC-restricted efficacy analysis, the lesion-adjusted model additionally included lesion size, lesion location, depth of invasion, and calendar period. For safety outcomes, lesion-adjusted models additionally included final pathology category, lesion size, lesion location, and calendar period. Additional surgery performed after ESD was not included in multivariable modeling because only 15 patients underwent additional surgery. This outcome was analyzed descriptively among patients with non-curative resection (Table 3). ‡ Multivariable estimates for post-ESD pneumonia and delirium were not calculated because only two and one events, respectively, occurred in the entire cohort, precluding reliable adjusted estimation. CCI, Charlson Comorbidity Index; CI, confidence interval; EGC, early gastric cancer; ESD, endoscopic submucosal dissection; OR, odds ratio.

## Data Availability

The data used to support the findings of this study are available from the corresponding author upon reasonable request, subject to institutional and ethical restrictions.

## Supplementary Materials

The following supporting information can be downloaded at: https://www.mdpi.com/article/10.3390/jcm15197373/s1, Table S1: Short-term safety outcomes stratified by CCI category and age group; Table S2: Covariate balance after 1:1 propensity-score matching; Table S3: Outcomes in the propensity-score-matched cohort; Table S4: Calendar-period sensitivity analysis.
