# Supplementary material for: Short-Term Safety of Gastric Endoscopic Submucosal Dissection in Patients Aged 80 Years or Older Under a Standardized Inpatient Post-Procedural Surveillance Pathway: Data from a 13-Year Real-World Cohort Study
Source: J Clin Med. 2026 Sep 23;15(19):7373. doi: 10.3390/jcm15197373 (PMC13665294; doi:10.3390/jcm15197373)
Supplement: Supplementary file 1 [file jcm-15-07373-s001.zip › jcm-4449345-supplementary.pdf]

Table S1. Short-term safety outcomes stratified by CCI category and age group

Outcome	CCI <3, <80 years (n=1020)	CCI <3, ≥80 years (n=143)	CCI ≥3, <80 years (n=716)	CCI ≥3, ≥80 years (n=142)
Post-ESD bleeding	16/1020 (1.6)	3/143 (2.1)	25/716 (3.5)	4/142 (2.8)
Post-ESD perforation	8/1020 (0.8)	0/143 (0.0)	5/716 (0.7)	1/142 (0.7)
Post-ESD pneumonia	0/1020 (0.0)	0/143 (0.0)	2/716 (0.3)	0/142 (0.0)
Post-ESD delirium	0/1020 (0.0)	0/143 (0.0)	0/716 (0.0)	1/142 (0.7)
30-day composite safety and healthcare-utilization outcome	61/1020 (6.0)	5/143 (3.5)	60/716 (8.4)	8/142 (5.6)
ESD-related readmission within 30 days	19/1020 (1.9)	0/143 (0.0)	17/716 (2.4)	2/142 (1.4)

Values are n/N (%). CCI, Charlson Comorbidity Index; ESD, endoscopic submucosal dissection.

Table S2. Covariate balance after 1:1 propensity-score matching

Characteristic	Matched <80 years (n=285)	Matched ≥80 years (n=285)	Absolute SMD
Female sex, n (%)	106 (37.2)	108 (37.9)	0.014
CCI, mean ± SD	2.42 ± 1.80	2.57 ± 1.82	0.085
CCI ≥3, n (%)	142 (49.8)	142 (49.8)	0.000
Antithrombotic-agent use, n (%)	12 (4.2)	12 (3.9)	0.018
Low-grade adenoma, n (%)	101 (35.4)	103 (36.1)	0.014
High-grade adenoma, n (%)	35 (12.3)	34 (11.9)	0.011
Early gastric cancer, n (%)	149 (52.3)	148 (51.9)	0.007
Procedure in 2020–2026, n (%)	191 (67.0)	197 (69.1)	0.045

Matching variables were sex, CCI, antithrombotic-agent use, final pathology category, and calendar period. Absolute standardized mean differences <0.10 indicate acceptable balance.

Table S3. Outcomes in the 1:1 propensity-score-matched cohort

Outcome	Matched <80 years (n=285)	Matched ≥80 years (n=285)	P value
Post-ESD bleeding	9/285 (3.2)	7/285 (2.5)	0.804
Post-ESD perforation	2/285 (0.7)	1/285 (0.4)	1.000
Post-ESD pneumonia	0/285 (0.0)	0/285 (0.0)	1.000
Post-ESD delirium	0/285 (0.0)	1/285 (0.4)	1.000
Emergency department visit within 7 days	3/285 (1.1)	0/285 (0.0)	0.250
ESD-related readmission within 30 days	9/285 (3.2)	2/285 (0.7)	0.065
Any hospitalization within 30 days	20/285 (7.0)	7/285 (2.5)	0.015

Values are n/N (%). P values are from exact McNemar tests for paired observations.

Table S4. Calendar-period sensitivity analysis

Outcome	Early <80 (n=721)	Early ≥80 (n=88)	Later <80 (n=1015)	Later ≥80 (n=197)	P for age×period
Post-ESD bleeding	16/721 (2.2)	3/88 (3.4)	25/1015 (2.5)	4/197 (2.0)	0.206
Post-ESD perforation	8/721 (1.1)	0/88 (0.0)	5/1015 (0.5)	1/197 (0.5)	0.411
Post-ESD delirium	0/721 (0.0)	0/88 (0.0)	0/1015 (0.0)	1/197 (0.5)	—
ESD-related readmission within 30 days	10/721 (1.4)	2/88 (2.3)	26/1015 (2.6)	0/197 (0.0)	0.042

Early period: April 2013–December 2019; later period: January 2020–March 2026. Values are n/N (%). Interaction P values are from lesion-adjusted models including age group, sex, CCI, antithrombotic-agent use, pathology category, lesion size per 10-mm increase, lesion location, calendar period, and the age-by-period term. Firth penalized logistic regression was used for sparse outcomes. Results should be interpreted cautiously. The age-by-period interaction for delirium was not estimable because only one delirium event occurred.
